# “The Drug Use Unfortunately isn’t all Bad”: Chronic Disease Self-Management Complexity and Strategy Among Marginalized People Who Use Drugs

**DOI:** 10.1177/10497323221083353

**Published:** 2022-03-24

**Authors:** Lisa M. Boucher, Esther S. Shoemaker, Clare E. Liddy, Lynne Leonard, Paul A. MacPherson, Justin Presseau, Alana Martin, Dave Pineau, Christine Lalonde, Nic Diliso, Terry Lafleche, Michael Fitzgerald, Claire E. Kendall

**Affiliations:** 1 School of Epidemiology and Public Health, Faculty of Medicine, 6363 University of Ottawa, Ottawa, ON, Canada; 2 C.T. Lamont Primary Health Care Research Centre, 152971 Bruyère Research Institute, Ottawa, ON, Canada; 3 Department of Family Medicine, Faculty of Medicine, 12365 University of Ottawa, Ottawa, ON, Canada; 4 Clinical Epidemiology Program, 10055 Ottawa Hospital Research Institute, Ottawa, ON, Canada; 5 Department of Medicine, Faculty of Medicine, 12365 University of Ottawa, Ottawa, ON, Canada; 6 Somerset West Community Health Centre, Ottawa, ON, Canada; 7 Centretown Community Health Centre, Ottawa, ON, Canada; 8 The CDSM Among PWUD Study’s Community Advisory Committee, 152971 Bruyère Research Institute, Ottawa, ON, Canada; 9 Sandy Hill Community Health Centre, Ottawa, ONCanada

**Keywords:** self-care, chronic disease management, drug use, self-medication, qualitative, community-based research

## Abstract

Self-management programs improve health outcomes and self-management is recommended for chronic conditions. Yet chronic disease self-management supports have rarely been applied to people who use drugs (PWUD). Thus, our objective was to explore self-management experiences among marginalized PWUD. We used community-based participatory methods and conducted qualitative interviews. Participants self-identified as having long-term and past year experience using non-prescribed drugs, one other chronic condition, and socioeconomic marginalization. We analyzed the data using reflexive thematic analysis. Although many participants considered drug use a chronic health issue, self-medicating with non-prescribed drugs was also a key self-management strategy to address other health issues. Participants also described numerous other strategies, including cognitive and behavioral tactics. These findings highlight the need for a safe supply of pharmaceutical-grade drugs to support self-management among marginalized PWUD. Self-management supports should also be tailored to address relevant topics (e.g., harm reduction, withdrawal), include creative activities, and not hinder PWUD’s agency.

## Background

Marginalized people who use drugs (PWUD) tend to experience complex health conditions characterized by chronicity and multimorbidity, as well as intersectional stigma and disparities related to social determinants of health (Biancarelli et al., 2019; Boucher et al., 2017; Dassieu et al., 2020; Degenhardt et al., 2013; Paquette et al., 2018). They have poorer health outcomes and lower life expectancy than the general population (Degenhardt et al., 2013). In Canada, the Expanded Chronic Care Model (E-CCM) (Barr et al., 2003) has been adopted for the prevention and management of increasing prevalence of chronic disease (Ontario Ministry of Health and Long-Term Care, 2007). However, despite calls for the treatment of substance use disorders as chronic conditions (Dennis & Scott, 2007; Kim et al., 2011; 2012; O’Connor, 2013), they are often not considered within chronic disease initiatives.

One domain of the E-CCM pertains to self-management (Barr et al., 2003), defined as “the tasks that an individual must undertake to live well with one or more chronic conditions. These tasks include gaining confidence to deal with the medical management, role management and emotional management of their conditions” (Adams et al., 2004). Recognizing that people with health conditions must practice self-management day-to-day outside of the healthcare system, self-management support aims to increase their confidence, motivation, knowledge, and skills to cope well with the impacts of illness (Liddy et al., 2014; Lorig, 2002). Self-management programs have been shown to improve health behaviors, health outcomes, and quality of life across chronic conditions (Allegrante et al., 2018), including for some disadvantaged groups (McDonald et al., 2004), and people with severe mental health issues (mhca Care Management Bulletin, 2015) or HIV (Boucher et al., 2019), common comorbidities with substance use disorders (Kendall et al., 2017). Regardless, disadvantaged populations have less access, lower participation, and greater attrition from self-management programs, despite having greater multimorbidity and worse outcomes (Mills et al., 2014), and there remains scarce research on supporting self-management among people with complex chronic diseases (Sevick et al., 2007).

One explanation for why self-management support has rarely been explicitly applied among PWUD is because of (mis)conceptions that they are either “deviants” who choose not to self-manage or that active drug use renders them incapable of self-management (Gowan et al., 2012; Szott, 2015). Prior studies describing self-care among PWUD have mainly focused on how people manage their drug use, especially the role of harm reduction practices (Boucher et al., 2017; Gowan et al., 2012; Greenspan et al., 2011), while other studies have focused on self-managing specific health issues (Holt & Treloar, 2008; Smith et al., 2014; Wilson et al., 2018). However, one qualitative study of 28 people in Florida, USA, considered self-care more broadly, finding that street drug users actively engaged in self-care practices such as eating healthy or exercising (Drumm et al., 2005).

Ensuring this community has access to adequate self-management supports should be a priority. Enhancing self-efficacy is the backbone of many self-management programs—that is, enhancing people’s belief in their capacity to perform self-managing behaviors (Bandura, 1977). Yet, while associations have been found between low self-efficacy and greater frequencies of drug use and relapse, there are also few interventions to improve self-efficacy among PWUD (Kadden & Litt, 2011). In addition, given PWUD’s multimorbidity and complex needs, generic chronic disease self-management programs may be more effective than disease-specific programs (Sevick et al., 2007). Regardless, to identify or adapt existing self-management initiatives and to develop new ones for PWUD—as with any under-researched population on a given topic—an understanding of their perspectives, experiences, and needs pertaining to self-management is first required (Lorig & Holman, 2003). Thus, the aim of this study is to explore the question: *How do marginalized PWUD self-manage their chronic health issues and drug use?*

## Methods

### Theoretical/Conceptual Frameworks and Methodological Approach

To accomplish our study aim, we conducted in-depth qualitative interviews and incorporated a holistic, person-centered approach that would highlight the agency of PWUD. Holistic approaches tend to be concerned with the “whole person,” attending to the “body-mind-spirit” (Frisch & Rabinowitsch, 2019). Patient-centeredness is often conceptualized in terms of provider-patient relationships, involving five dimensions: “biopsychosocial perspective; ‘patient-as-person’; sharing power and responsibility; therapeutic alliance; and ‘doctor-as-person’” (Mead & Bower, 2000); though we employ the term “person” instead of “patient” because it “directs energy to those managing their conditions in their own way, as it were ‘outside’ the health system,” which better includes marginalized groups and thus health equity concerns (Pulvirenti et al., 2014). Holistic and person-centered approaches are well-aligned (sometimes even paired), and notably, both have been conceptualized as important principles for optimizing self-management/self-care (Pulvirenti et al., 2014; Pyles, 2020). However, past research has found patient-centeredness to be limited within clinical chronic disease self-management initiatives, especially with respect to valuing patients’ experiential knowledge of self-management strategies or ensuring meaningful shared decision-making (Kendall et al., 2011; Rogers et al., 2005). Further, providers may incorporate more narrow interpretations of self-management than patients and experience organizational constraints to implementing holistic, patient-centered care (Rogers et al., 2005). Acknowledging these issues, as well as the likelihood they are exacerbated among marginalized groups, we aimed to understand PWUD’s own conceptualizations of their illness and self-management experiences.

Accordingly, we also incorporated a transformative framework meant to amplify the voices of marginalized groups toward addressing social justice (Creswell & Poth, 2018; Mertens, 2007). The transformative paradigm highlights the importance of interrogating privilege and power structures and attending to cultural complexity (Mertens, 2007). We recognized the disadvantaged social positions of our participants and the need to better understand their complex lived realities—especially due to our focus on PWUD who are socioeconomically marginalized, but also their identification with other marginalized groups (e.g., racial or gender minorities). This led us to focus on creating a non-judgmental space where participants’ own preferences and most important concerns could come to the forefront. This information is pertinent because, while patients in general may be recognized as experts on their own self-management (Lorig, 2002), marginalized groups are often left out of decisions that affect them.

Complementarily, we established a community-academic partnership through the pragmatic use of community-based participatory research (CBPR) (Israel et al., 2010; The SAGE Qualitative Research Kit, 2007). CBPR emphasizes collaboration and meaningful participation of community stakeholders, with goals of co-learning and taking action for social change (Flicker, 2005). CBPR methods were critical for this study because expert consensus has highlighted the importance of meaningfully engaging people with lived experience in self-management support initiatives, as well as enhancing health equity in the field (Mills et al., 2016). Thus, improving consultation with marginalized groups can help to meet these goals and increase the relevance of self-management supports.

Overall, we anticipated that some marginalized populations’ experiences may not reflect the current self-management frameworks commonly applied to general populations, including by not incorporating a holistic and person-centered approach that attends to the complex needs of these groups. We considered this exploratory study to be a first step toward determining which aspects of self-management initiatives may or may not be appropriate or useful for marginalized PWUD, with the larger purpose of advancing knowledge that can address the health inequities this group experiences.

### Research Location and Team

The study took place in Ottawa, Ontario, Canada, and was approved by Bruyère Research Institute (M16-19–027) and University of Ottawa (H-10–19–5175) Research Ethics Boards. In accordance with CBPR practices, we engaged community members throughout the research process. The lead author drew on prior experience working in a CBPR capacity with the intended population and enlisted key community members with lived experience of drug use issues, other chronic health issues, and socioeconomic marginalization, and who were well-known and respected among the local community. Due to pervasive stigma and prior negative experiences, marginalized PWUD often distrust care providers or other authority figures; hence, well-connected and trusted community members were crucial in this study (Newcombe, 2013).

At the onset of the study, we engaged one community member with lived experience as a Community Research Coordinator. This individual had extensive experience from prior research studies in this type of role, which in this study involved identifying four other community members with lived experience to participate on a Community Advisory Committee (CAC) and helping to coordinate and facilitate their activities. The primary roles of CAC members were to provide guidance on study methods, develop research tools, conduct recruitment, support analysis and interpretation of findings, and support knowledge translation. We selected community members based on: (1) diversity in past research training or experience; (2) representation of diverse characteristics and experiences among the study population; and (3) current engagement in various capacities with community organizations. To promote consistency in this study, they also received (new or refresher) training in research ethics, qualitative methods, recruitment, data analysis, and knowledge translation. CAC members signed confidentiality agreements and received honoraria for their work.

### Interview Guide Development

We held CAC meetings to create a semi-structured interview guide. Questions broadly investigated how people’s chronic health issues and drug use influenced each other, as well as their motivations, needs, and experiences in regard to personal self-management practices and receiving self-management supports. Recognizing the breadth and complexity of the topic, CAC discussions included time focused on ensuring the language was appropriate for the community. For instance, we adjusted the phrasing of questions to avoid using only the term “self-management,” instead asking how people “manage” their long-term health issues or using the more familiar term “self-care.” Our key questions were broad, such as “What do you do on a day-to-day basis to manage your chronic health issues?” We incorporated a funnel approach in which open-ended questions were asked initially to encourage free-form conversation on most topics. If certain questions of interest did not arise naturally, they were asked explicitly before the end of the interview (Morgan, 1996). We reviewed our interview process throughout—adapting questions organically to address participants’ challenges in interpretation and new issues they highlighted as important to self-management—and overall ended up using a more unstructured interview style to obtain the most relevant information. Our interview guide is included as Supplemental Material.

### Sampling and Recruitment

We selected eligibility criteria through CAC discussions, specifying that participants must self-identify as having: (1) long-term experience using non-prescribed drugs (i.e., illegally obtained drugs or prescription drugs used not as prescribed, beyond exclusively cannabis), including in the past year; (2) one other long-term (chronic) health issue; and (3) current financial difficulties (i.e., to meet our definition for “socioeconomic marginalization”). While PWUD also experience other types of marginalization, we chose to focus on those who were socioeconomically marginalized (Richardson et al., 2015). This was mainly due to the CAC’s concerns about potentially grouping PWUD who struggled with financial instability with those who did not, as they anticipated unique self-management experiences and needs. Additionally, this focus helped us unify our sample around one type of marginalization. We aimed for a purposeful sample of participants using the sampling strategy of maximum variation in order to identify a diverse range of perspectives (Palinkas et al., 2015). The CAC determined as the most relevant factors: age, sex and gender, sexual orientation, ethnicity, drug use characteristics, common health issues in the community, common marginalizing experiences (e.g., housing instability, sex work, incarceration), and people’s level of engagement in services.

We used a peer-led, street-based recruitment approach, which has successfully been employed in prior studies among this community (Boucher et al., 2017). With community input, we identified “hot spot” locations for in-person recruitment, mainly focusing on areas of the city near our planned interview locations to facilitate accessibility. On each data collection day, one of three community researchers led the recruitment process. This procedure capitalized on the community researchers’ trusted position in the community to facilitate the lead author’s introduction to and rapport with potential participants. Individuals recruited were typically scheduled to participate in an interview within a short timeframe to reduce no-shows. We recruited participants between December 2019 and March 2020, at which time data collection was suspended due to the COVID-19 pandemic.

### Data Collection and Analysis

We conducted 15 individual, in-person qualitative interviews with recruited participants, scheduled so that we could conduct ongoing, iterative analysis, refining our recruitment strategy and interview guide throughout data collection. Interviews took place at four community-preferred sites, including several community-based organizations frequented by PWUD in our setting. The lead author obtained written informed consent and conducted all interviews. We administered a brief questionnaire to collect sociodemographic information from participants, with results summarized descriptively in Supplemedntal Table 1. Participants were compensated CAD 30.00 for their time.

All interviews were recorded and transcribed verbatim, with hand-coding and NVivo software used to code the transcripts (QSR International Pty Ltd, 2013). Audio recordings of the interviews had a mean length of 1 hour and 13 minutes (range = 39 minutes to 1 hour and 49 minutes). The lead author (trained in multiple research methods through psychology and epidemiology) fully coded all transcripts, while a qualitatively trained medical sociologist coded half of the transcripts, and a community researcher coded two transcripts and reviewed codes of all prominent categories to provide cultural interpretation. We used an inductive approach, with all codes and themes generated from data analysis rather than identified in advance. Major and minor themes, and relationships among them, were brought to discussions with both academic and community researchers, and further adjustments were made to reflect insights gained.

We used the qualitative research approach of reflexive thematic analysis (Braun & Clarke, 2006, 2019a, 2020). This involved considering both manifest and latent content during data analysis, with a focus on identifying the patterns and threads (themes) across the data. We also adopted constant comparison and line-by-line coding to enhance validity of our findings (Creswell & Poth, 2018). Aligning with reflexive thematic analysis, each reflexive iteration allowed us to increase our focus and progressively make meaning (Srivastava & Hopwood, 2009). A focus on achieving data saturation was not required nor conducive to this type of analysis (Braun & Clarke, 2019b). Rather, we employed the concept of “information power” to determine that the sample data contained adequate information to provide new insights relevant to our research question (Malterud et al., 2016). In particular, we had sufficient information power due to the very strong quality of the interview dialogue, bolstered by the interviewer’s extensive familiarity with the population and research topic, as well as our incorporation of theory in the study design and a highly purposive recruitment strategy that led to specificity in the sample.

To further enhance quality and rigor in our methods, we considered Guba’s (Guba, 1981) four criteria for trustworthiness: credibility, transferability, dependability, and confirmability. In addition to constant comparison and line-by-line coding, we used the following strategies during data collection and analysis to meet the criteria: tactics to enhance or detect informant honesty, recording iterative reflective notes, debriefing between the interviewer and experts on and off the research team (including people with lived experience), and using thick quotes in dissemination to allow the reader to make their own judgements (e.g., credibility); collecting and reporting rich contextual details (including on setting and participants) to allow assessment of whether findings may be applicable to other contexts (e.g., transferability); documenting challenges and discussions to maintain a decision trail, and reporting our methods in detail to facilitate repeatability (e.g., dependability); and acknowledging personal biases and assumptions (including examining our social positions and power), and providing in-depth description of shortcomings (e.g., confirmability) (Noble & Smith, 2015; Shenton, 2004).

## Results

### Portrait of Self-Reported Chronic Health Issues

When asked to describe their chronic health issues, participants detailed a wide range of concerns, including mental health issues, primarily pain-related issues, infectious diseases and their comorbidities, and other physical health issues (e.g., colitis, chronic obstructive pulmonary disorder). The most frequently reported chronic physical health issue was pain, which was diverse in terms of severity and source or type (e.g., feet, back, migraines, arthritis/joints). A majority of participants also reported infectious diseases (e.g., HIV, hepatitis C). While all participants reported having current or previous mental health symptoms or conditions, only approximately half described these as chronic. The most commonly discussed mental health issues included depression, anxiety, and post-traumatic stress disorder. Many participants mentioned having experienced significant symptoms, including suicidal thoughts or attempts. Participants often described mental health issues in terms of having too much “stress” or being “overwhelmed,” rather than as specific diagnoses (although sometimes they did both). Similarly, they often described these issues as directly related to previous or current negative (including traumatic) life experiences.

Extensive multimorbidity and comorbidity were common, including issues related to aging or long-term disease progression (e.g., with HIV). Participants often mentioned a number of acute and recurrent symptoms and diagnoses such as frequent infections that, in their entirety, were experienced as chronic health concerns. For instance, in response to being asked to list chronic conditions, one participant said: *“Like, I go through a series of colds and like, flulike symptoms, which makes me overall pretty paranoid with other viruses that might be out there. So I get kind of confused, because 1 day I’ll be fine, the next day I won’t be fine...”* Participants had trouble separating their descriptions of acute versus chronic health issues, partly because the former often progressed to the latter. Participants typically did not prioritize one health issue over others (including drug use), instead thinking about their health and self-management in a holistic way.

All participants had many years of experience using drugs and most described a preference for a certain type of drug—usually either “uppers” or “downers” (i.e., crack/cocaine or crystal meth vs. opioids)—although they were still polydrug users. Current or past injection drug use was common, as was smoking, snorting, and ingesting pills. As for legal “recreational” drugs, several participants reported regular alcohol use, a substantial number used cannabis, and the majority smoked cigarettes. While cannabis has only been legal in our setting since October 2018, participants were using it well before this time.

All but one participant considered their non-prescribed drug use to be a chronic health issue, even those who felt they were currently controlling their use well but had more issues with it in the past. As one participant responded: *“I do, because look if somebody’ll put it in front of me and I can do it, I would do it, I will do it. … I do see it as chronic because I can never imagine myself being completely abstinent. I don't really want to be completely abstinent.”*

However, some participants clarified that they thought of their drug use as different from their other chronic health issues, for instance describing it as more episodic or not as much of an issue if it was well-managed. Further, some participants considered their drug use a chronic health issue intricately related to one of their other health issues (e.g., comorbidities), especially mental health or chronic pain: *“So that’s another chronic health issue is just the addiction and the like depression*… *Yeah, I get a phone call and it's like I don't want to answer the phone, it feels overwhelming. I have to be high to do anything.”*

Supplemental Figure 1 provides a summary depiction of participants’ overall health concerns, highlighting the most common or impactful examples they discussed.

### Themes Generated From Data Analysis

Our analysis identified two overarching themes depicting participants’ lived experiences of self-managing their chronic health issues including non-prescribed drug use, each with four sub-themes: (1) Participants’ non-prescribed drug use interrelated with chronic health issues and self-management in complex ways; and (2) Participants employed many personalized strategies similar to those in generic self-management initiatives.

### 1. Participants’ non-prescribed drug use interrelated with chronic health issues and self-management in complex ways

Participants’ experience of their drug use as a chronic health issue was complicated by the fact that using drugs was one of their key strategies for self-managing their other chronic health issues. Other complications for participants’ self-management included the detrimental symptoms they experienced when they did not have their drugs or when they were using in unstable ways. Participants recognized these complexities and attempted to stabilize their drug use through using harm reduction strategies.

#### 1.1 Drug use as self-medication for other health issues

Self-medicating with non-prescribed drugs was the crux of participants’ self-management regimen for their chronic health issues, and often the first practice they mentioned, as demonstrated in this exchange:

Interviewer: What do you do on a daily basis to manage those different health issues?

Participant: Well, it depends on which health issue I’m having that day, I guess. …For the arthritis, I will tend to find some purple [fentanyl], and usually, when I do the purple, it’ll relieve a number of symptoms that I’m having, such as like, if I’m having cold sweats or if my muscles, joints are hurting... Which, then for the crack cocaine or the cocaine or whatever…that helps like, my mental issues like, if I’m feeling depressed or if I don’t want to think at all…

In describing how they used non-prescribed drugs to treat mental and physical health issues, participants chose certain drugs to help with different symptoms. Opioids were most frequently discussed and considered helpful for pain (especially physical pain, but also emotional pain). Stimulants were mainly described as useful to treat mental health issues, although some participants noted they could help with physical health issues too by allowing them to forget for awhile. As a participant with anxiety explained:

Participant: I’m moving at a sluggish pace because I’m just – I don’t have what I would almost call my medicine, right, at this point.

Interviewer: OK, makes sense. So, what would that be then? Like, which drugs…

Participant: Oh, crystal meth is what I normally use. …it’s what works best with my brain chemistry. …the drug use alleviates a lot of the anxiety, right? …The drug use unfortunately isn’t all bad. It gives you confidence and gives you motivation sometimes. Gives you the energy you need to go through your day and get–accomplish what you need to accomplish.

Participants also described their self-medication practices in ways that indicated their goal was to mitigate their symptoms rather than to get high: *“Because I wasn't addicted to the stuff... I was doing it on my own terms, I’d do a shot and if I felt the shot like I wouldn't do it for 2 days because…sometimes I don't get a migraine every day, so it's just when I need it...”*

Participants reported that cannabis was the most beneficial drug they consumed as self-medication. They noted that cannabis helped with managing symptoms of a wide variety of health issues, in particular gastrointestinal issues, chronic pain, sleep problems, and emotional dysregulation. One participant described the substantial positive influence of cannabis on his ability to self-manage:

…weed has probably played the biggest role in my life, it was for medication… it just helps me a lot with my mental health and my physical… I will forget to smoke weed because I don’t crave it like that. …then I do [smoke] and all of a sudden it’s like OK, let’s get a job… [it] makes me a lot better at taking care of myself. …I’m like dude, you should probably drink some water, that type of thing. …it takes me out of the part of my brain that I let the negative voice happen… and then I can focus on taking care of myself.

However, participants noted having trouble affording cannabis in addition to affording their other drugs and basic needs. Furthermore, many preferred using non-prescribed drugs to self-medicate over the use of mental health medications. For instance, one participant who used opioids to help cope with depression and difficulty sleeping, described the negative experiences she had had with prescription medications:

Participant: …I don’t like to take anti–depressants, I don’t like Seroquel and all that.

Interviewer: So you’re not on any mental health medications?

Participant: Valium and all that – clonazepam or clonidine or…

Interviewer: Okay, have you tried any of them?

Participant: Yeah, I don’t like any of them, no, they make me feel worse.

#### 1.2 Lacking access to drugs interfered with self-managing and health

Participants expressed detriments to their self-management abilities and health overall when they did not have consistent access to their drugs. Typically, this pertained to opioid users and the challenges of physical withdrawal, which was compounded by the exacerbation of chronic pain among some participants: “*I'm always having a bad day. My legs already hurt right? But just the level of hurt right? So nothing in my system–like I'm almost in tears.”*

Participants also explicitly noted that withdrawal interfered with self-managing their other health issues: “*And I wouldn't change my bandages unless – if I was dope sick I’d leave it on forever. Because it hurts so much.”* They also discussed the importance of having strategies to avoid withdrawal: “*Like I always have to plan ahead a little bit, every day, to make sure that in the morning that I’m going to have enough [drugs]…so at least I can get a start, start my day…”*

Similarly, participants who used stimulants described experiencing negative symptoms when they did not have their drugs. As one crystal meth user explained, the most challenging aspect of their drug use was when they lacked access to it:

Interviewer: Do you get any symptoms from the drug use that are negative? You already mentioned like, positives from it…

Participant: Well, it’s only if I don’t have it. Like, today for example, like, I’m low energy, I’m not really feeling like myself completely, you know?

#### 1.3 Desire to improve stability of drug use to improve health

Participants often noted that their self-care goals included stopping or better managing their drug use. A few described how they had accumulated worsening chronic health issues over time or as they aged, and subsequently their drug use became more difficult to sustain without further worsening their health, as one participant explained:

And then the emphysema is what actually was my brick wall because I couldn’t do it anymore. I couldn’t smoke crack anymore, I couldn’t smoke cigarettes anymore, I couldn’t walk across the street anymore without, you know [shortness of breath sounds]. … With the opiates, it took me longer to stop… I think it had a lot to do with the fact that I really messed up my veins.

Some participants described how drug use interfered with self-managing their other health conditions, especially if they were experiencing unstable drug use. As one participant noted:

A lot of the health issues I have is from HIV and the side effects, but I think the using of the illicit drug isn’t helping. …When you’re using the drugs, your immune system is more low. I’m not eating because you don’t want to eat. I try to take multi-vitamins and vitamin C, but then if you don’t have any substance in you they’re just not absorbed. …So, I mean, I know all these things, but it’s hard to practice when you’re [using].

Thus, participants typically desired to better control their drug use with the aim of improving the prognosis of other health conditions, as one participant described: *“But [hepatitis C]’s the one I'm worried about now because I don't want to drink as much as I want to. I mean, I want to drink but I can't. I have to watch my liver enzymes…”*

Participants also noted having made progress with respect to using drugs in less detrimental ways, such as how replacing the drugs that caused them more problems with other drugs could be beneficial for managing their health overall:

I don’t want to go through life without ever catching any kind of a buzz but I don’t want it to consume me and cost me everything and kill my health… So a couple of drinks here and there, eat a pot cookie, I’m good with that, you know? That’s about where I’m finding myself now. …now that we’re talking about things like the psilocybin and the edibles and stuff, like I really should be focusing more on that. …you know, just give you a little lift and be safer and be beneficial in other ways too, like therapeutic value…

Furthermore, participants were aware of and constantly weighed the pros and cons of their drug use with respect to their health, making efforts to balance these. Most did not consider complete cessation of drug use to be a solution, as one participant with extensive multimorbidity expressed:

Well, I’m kind of like, confused with like how long my drug use is either going to let me live versus the healthcare that I’m getting. If I were to quit drugs would I live longer, or is it the drugs that I’m taking now with the care that’s making me live longer? Because you know, if you do something for a long time and then you take it away, you – your body can either like – need it, or your body can get really, really sick without it…

#### 1.4 Employing harm reduction practices to manage drug use

Overall, harm reduction was clearly an important part of PWUD’s self-care regimen, and central to managing their drug use. Yet given the context of the longstanding overdose crisis in our setting (Public Health Ontario, n.d.), it was not surprising that much of the harm reduction practices they discussed pertained to reducing risk of overdose. These practices were ultimately about survival, as many had overdosed themselves or lost loved ones to fatal overdose, yet their worries about dying and grief interfered with their mental health. People described using supervised injection services, keeping naloxone on them, using with other people nearby, using pharmaceuticals or avoiding street drugs, buying from trusted drug sellers, avoiding fentanyl, and testing drugs using test kits or by taking small first doses.

Other key harm reduction practices included using in moderation, tapering drugs to avoid or reduce withdrawal effects, avoiding certain drugs, replacing drugs with less harmful (or even beneficial) ones, using sterile equipment, using less harmful routes of administration, and saving doses to avoid having to go without at future times. For example, as one participant described:

…if somebody gives it to me for free, okay, sure I’ll do a little puff and I won’t do it until after I’ve had lunch kind of thing, whereas before I was doing crack 24 hours a day almost. … At least now I’ve slowed down and I actually have time to think…like that’s why I think it’s better now, like that’s why my creativity has really been coming back more bit by bit…

Many participants also noted how a safe supply of pharmaceutical-grade drugs would improve their ability to practice self-care. Although this desire was most prominent among people currently obtaining opioids from the street, participants using other types of drugs also described how a safe supply would not only reduce their likelihood of death, but help to address their challenging financial situations, thus allowing more time to focus on their health issues:

Interviewer: …if you could get any support or service that you’re not getting, what would that be?

Participant: It would be something like safe supply. … Now that I see some of my friends on it I’m like, jealous, cause they have more time to figure out their problems and they have more times to themselves because they’re not sitting there chasing the drug all the time or chasing after money…

Another participant described how safe supply could be a starting point from which marginalized PWUD could build the foundations necessary to develop self-care practices:

But I think ultimately one of the first ways that we can have people start getting some security, like right now if you're on the street and you're hooked and you're homeless and all that, if all you could get was a safe supply of clean pharmaceuticals that wasn't going to kill you, that doesn't fuck with your mind and neurology and biology the way that this other bad drugs do, it would be a good start. Because then maybe they could start thinking oh well, now that I'm secure in this, now can I think about getting a place, now can I think about seeing a doctor, now can I think about addressing this health issue and that health issue...

### 2. Participants employed many personalized strategies similar to those in generic self-management initiatives

Participants described many different types of strategies, which they often used in a holistic manner, not only to cope with current health issues but also for preventive purposes. As one participant described, cultivating an extensive self-care regimen had highly positive effects on her health:

…now I have a little skin care routine…I make sure I get enough sleep. I’m choosing, more wisely what I eat. I’m more thoughtful and mindful about, what I want to do to not only protect my health and improve it but to prevent stuff. … And the more that I’m developing self-care habits, the proof is in the pudding because the difference can be felt and it’s amazing to me.

#### 2.1 Most commonly recognized self-management behaviors

Several of the strategies participants described are the most well-recognized self-care strategies in general, aligning with people’s basic needs, and commonly addressed within self-management programs, such as nutrition and physical activity (Lorig et al., 2013). A few also discussed the importance of sleep, as well as personal and living space hygiene. Whether participants prioritized certain activities over others depended on which ones helped relieve the symptoms of their specific chronic health issues. For instance, nutrition was central to a participant with colitis, while monitoring sleep was a key strategy for a participant with bipolar disorder. However, many participants noted that they struggled to perform these strategies. For example, one participant said: *“We don't eat proper anything.”* Others mentioned how obtaining food prior to their drugs was a self-management strategy: *“I'll make sure, like, I have food before drugs.”*

As for physical activity, several participants mentioned doing yoga or going to the gym. Participants also made it clear that they obtained much exercise through walking to conduct their typical daily activities, including obtaining their drugs: *“…cause you got to think about if you’re looking for something, you’re stuck downtown, you’re walking and you’re walking…you walk a lot. I walk like all over the place…”*

#### 2.2 Cognitive and behavioral strategies, especially creative activities

Participants employed many cognitive and behavioral strategies to manage their health issues, including techniques for relaxation (e.g., deep breathing, meditation, visualization, gardening, knitting, hot bath), mindfulness, positive thinking, distractions, being in nature, keeping busy, developing a routine, reflecting on patterns between behaviors and symptoms, prioritizing, committing to an action, acknowledging limitations, and taking responsibility. For example, one participant described her relaxation process in detail:

So if I feel like I’m getting too stressed out I imagine with every breath in I take, I am breathing in, taking all the toxins and all the stress I have, when I’m breathing it in I imagine that I’m storing them in my inside when I breathe in. Hold it in for eight counts, and then I exhale all that garbage out as hard and as loud as I can. It works.

Similarly, focusing on spirituality was an important strategy among participants, especially to help manage mental health issues. For some, this involved following the practices of organized religion or engaging with certain spiritual groups, whereas for others it was described as a more personal or introspective journey. Some participants reported regular engagement with spiritual activities—*“…my typical day is, I pray a lot… So, that’s part of the self-care, a little spirituality.”*—while others mentioned recent attempts to further embrace these kinds of strategies. Indigenous participants highlighted cultural-spiritual traditions as meaningful for their mental health and desired more opportunities to engage in these. As one participant described, the ability to spend time in nature would be beneficial: *“Yeah, it would be so good to get out of the city once in a while, stuff like that, like go sit by a lake or a river or ocean. You know, burn and cook things with a fire like my grandfather used to. I would love that but I…I have no access.”*

A few participants also discussed the importance of time management. While some noted how they mainly focused on the present day, one participant explained how planning her days in advance led to improvements in managing emotional symptoms:

…my whole life did everything by the seat of my pants, couldn’t plan 30 seconds ahead. … But now I live by my calendar. … And it helps me psychologically to write things down, like this is what I’ve got to do tomorrow. …and then there’s way less stress because like I know what I’m doing, when I’m doing it. And I still can be spontaneous and flexible but I just really am enjoying how it makes my life easier and I’m not as anxiety ridden as I ever used to be all the time about stuff… And so I feel good about that because I think that’s part of self-care is managing my time so that I’m not freaking out all the time…

The most prominent type of cognitive and behavioral strategies involved having creative outlets to cope with the negative emotional effects of health issues. Many participants noted that they engaged in (or wanted to engage in) arts-based activities, including music, fine arts, visual arts, performance arts, decorative arts, cosmetic arts, and making handmade crafts. As one participant said: *“Music… It takes a lot of stress away.”* They also reported how these practices were beneficial and motivating for better managing drug use:

And I’m just in a transitional phase. It’s really weird because I’m starting to not abuse drugs again. And getting back into music… I’m trying to rediscover parts about me that I still want from before I used, you know? Cooking, like the cooking thing, I was working in fine dining.

However, participants whose drug use was more unstable tended to have trouble engaging in these desired activities:

Oh yeah, I miss playing my guitar all the time and tattooing and drawing and stuff. … When I quit doing drugs I need to be doing something or I’ll be out making money to get drugs, getting drugs, or getting high. Like my life revolves around dope. … That relieves a bit of stress, and I’ve been playing for over 30 years right?

Participants also highlighted how these creative activities went beyond simply managing emotional dysregulation, as they were often central to their future goals and to cultivating a fulfilling life: “*I’m an artist and I really want to get back into…that sort of thing. And I just want to like, launch back into a lifestyle that I can be proud of.”*

#### 2.3 Seeking out healthcare resources

Several participants noted how they felt the need to be proactive in monitoring their health issues and were aware of supports available: *“I know where to go and how to get supports if I need them. Just pick up a phone or just walk into a place and say hey, I could use some of this or that.”*

Other participants were less familiar with how to obtain supports, but still indicated that they knew it was important to seek them out: *“I know I need somebody to help right? So I can't do it on my own obviously, or it would be done so–and I am going to–I got to start taking care of myself better.”*

Some participants had not been well engaged in care but as their health issues worsened they had begun to obtain support: *“Well right now, I’m getting scared that I’m going to end up in the home, so that’s what’s motivating me, I guess, is fear. … I just finally got a family doctor last week. … I did about 10 years without one.”*

In addition, participants often sought formal treatment to help manage their chronic pain, mental health issues, or drug use, with limited success. Many had tried opioid agonist therapy, yet some were unhappy with it and tried to wean themselves off. Participants unwilling to use opioid agonist therapy noted limited options: “*And I’ve been to, you know, [a clinic] a few times to see the doctors there. …just to talk about different alternatives. But you know, their alternatives, it’s either methadone or Suboxone, and I don’t like them both.”*

More rarely, prescriptions were available to treat other types of drug use, including alcohol, nicotine, and other stimulants. Sometimes participants obtained prescriptions second-hand through other people.

Pain-related supports were especially hard to come by for PWUD, and participants reported this as a priority gap in managing their health. One participant highlighted how better access to pain medication would help him maintain a more consistent schedule:

But if I had a script I’d take them normally, and then I’d have them on schedule because when I buy them I don’t get that much and I don’t have enough money to get them, so I have to space them out. And it’s hard to say which migraine is going to be the worst… so I’d rather try to get a script so I can like take them regularly… so I can live my life.

#### 2.4 Adhering to healthcare guidance

Some participants highlighted the importance of adhering to healthcare regimens for managing their chronic health issues, including taking prescription medications as directed or consistently attending appointments with healthcare providers: *“One pill every day for ever... But I go to the doctor regularly and get bloodwork done and they do my CD4, CD8 and my liver and everything like that. … It's important to me to know what my counts are because I need to know. Like, my viral load for HIV is undetectable. Which is good.”*

Participants also described having multiple medications for their different health issues, and the importance of having a routine for remembering to take them: *“I mean, it’s – you can remember taking your medication every day and that but now I'm on medication in the day time; I'm on medication at night time, medication at supper time. So like it’s – I got to be on a regular base with my medication.”*

Regular engagement with providers was also a strategy for preventing further health issues, especially among those who were thinking about aging. Some participants noted how they followed non-pharmacological instructions from their providers, such as improving nutrition and physical activity, partly because they wanted to avoid the need for more medications:

*“Because in my mind I feel that has a little impact because my bones are really hardening this year. And then also, the cholesterol, it’s been like over 20 years now that I’ve kept just borderline, for 20 years, on diet and exercise. …it was recommended to diet and exercise first. The doctor. A lot of it came from the doctor, plus I don’t want to take a whole bunch of pills too, well nobody does.”*

Still, at times participants mentioned having difficulty remembering to take medications or attend appointments, suggesting that their drug use or other complexities of their health and social issues interfered with maintaining a routine.

## Discussion

In this study, we addressed a knowledge gap related to chronic disease self-management among marginalized PWUD. Our approach involved moving beyond a disease-specific perspective to take a holistic, person-centered view of how PWUD self-manage their chronic conditions, highlighting the interplay between managing drug use and other health issues. This distinguishes our work from past research on self-care among PWUD, which has focused on either single health issues (e.g., mental health, chronic pain) or more commonly on drug use or harm reduction only. Furthermore, using the transformative paradigm and a CBPR design to gather the unique experiences of PWUD was critical to ensuring that we emphasized self-managing as a self-defined process, recognizing PWUD’s decisive agency and aiming to enhance power and equity for this marginalized group (Kendall et al., 2011). This approach led us to appreciate participants’ broad views on health and self-management, which extend beyond any single health condition or any single reason for using a particular strategy (e.g., several referred to “taking care of myself”). Such non-disease-specific views are consistent with the holistic nature of prominent strategies they highlighted (e.g., creative activities) and demonstrate the need to embrace more person-centered and whole-person approaches as a basis for self-care initiatives (Frisch & Rabinowitsch, 2019).

Our findings demonstrate the enormous multimorbidity and diversity of chronic health issues that PWUD experience, most notably various forms of chronic pain. Our findings also highlight the complexity surrounding PWUD’s self-management experiences, foremost of which was the use of non-prescribed drugs to self-medicate chronic health issues. However, this was not a simple, linearly beneficial relationship, as participants also expressed how their drug use itself was a chronic health issue and they desired to manage it better. Still, often the most challenging aspect of their drug use was managing the symptoms that resulted when they were unable to consume drugs. For these reasons, it is not surprising that while most participants considered their drug use to be a chronic health issue, they described it differently from their other chronic conditions. While there is quantitative evidence that substance use disorders are more likely to be chronic (Fleury et al., 2016), our qualitative CBPR approach provides a more nuanced understanding of what this means in the daily lives of PWUD. Overall, the relationship between drug use and other conditions was bidirectional, highly complicated, and always changing, with other conditions sometimes leading to worsened or improved drug use, and drug use sometimes worsening or improving other conditions.

Participants highlighted many self-management strategies, with the types of strategies chosen demonstrating the importance of agency in self-management decisions. Self-medication was particularly prominent in their accounts, mainly including the use of opioids, stimulants, and cannabis to mitigate symptoms from mental health conditions, chronic pain, and gastrointestinal or sleep-related issues. The self-medication hypothesis specifies that people use non-prescribed drugs to treat mental health symptoms (Khantzian, 1997). Yet our data highlight that participants used drugs for a larger range of ailments, especially an array of primarily pain-related issues. As other research has found, PWUD may trust their ability to self-medicate more than medical doctors (Smirnova & Owens, 2017) or they may lack access to healthcare and pain medication (Dassieu et al., 2019b; Voon et al., 2014). In addition, many of the self-management strategies participants described are activities typically classified as harm reduction practices. This corroborates previous work demonstrating the overlap between harm reduction and self-management practices among PWUD (Boucher et al., 2017). In the present study, however, PWUD’s harm reduction strategies were greatly driven by the overdose crisis.

Few participants detailed using the most well-recognized self-care strategies such as nutrition and physical activity, topics that are typically a focus in self-management programs (Lorig et al., 2013). Other strategies did map onto common self-management program topics, such as cognitive and behavioral strategies, seeking out healthcare resources, and adhering to healthcare guidance. However, much of the participants’ self-management involved strategies that are unlikely to be included or accepted within generic self-management programs, most notably self-medication with non-prescribed drugs, but also harm reduction practices and managing aspects of drug use such as withdrawal. Thus, the self-management needs of PWUD may not conform well to the information promoted in typical chronic disease self-management programs, and self-management supports should ideally be tailored to include the most relevant topics for this population. Still, generic programs often include skill-building activities that focus on improving self-efficacy and involve some tailoring to individuals, aspects which may make them useful for PWUD. Hence, we suggest that if these supports are provided, a peer-led approach would enhance comfort with discussing the issues identified in our study, as peer support is particularly helpful for disadvantaged and stigmatized groups (Sokol & Fisher, 2016). For instance, one qualitative evaluation of a self-management program with people living with HIV highlighted the need to focus more on mutual aid and collectively defining user needs (Kennedy et al., 2007). Furthermore, such supports for PWUD should not be strictly focused on chronic health issues, given the prevalence of acute health issues which may not only take precedence but also be experienced as chronic or become chronic if unaddressed. Additionally, the benefits of creative pursuits for many PWUD speaks to the need to attend to such activities within self-management supports, and perhaps suggests a role for improving access to art therapy and music therapy to support PWUD.

Our results also highlight that self-medicating chronic pain was a major force in perpetuating drug use. While most participants felt well supported for managing their infectious diseases, accessing adequate pain management supports was a clear gap. Substance use and mental health supports were also inadequate. To improve their capacity to self-manage, PWUD need improved access to chronic pain, mental health, and substance use supports. While multidisciplinary care may be the gold standard for these complex health issues, such supports are rarely accessible to socioeconomically marginalized groups. For PWUD, especially those with highly unstable drug use, these services must also be low-threshold—meaning they have taken steps to reduce barriers that obstruct marginalized groups from accessing (e.g., through drop-in or outreach provision, anonymity and non-judgement) (Islam et al., 2013). Furthermore, providing a safe supply of pharmaceutical-grade drugs that meet people’s needs is critical as a first-line measure, for numerous reasons. These reasons reflect recent findings from an evaluation of an opioid safe supply program in Vancouver, Canada, including reducing overdose risk, improving health overall but especially management of pain-related issues, and mitigating financial struggles (Ivsins et al., 2020). On the other hand, current opioid agonist therapy options are insufficient to meet the (self-)medication and life-saving needs of many PWUD, especially if living with chronic pain (Dassieu et al., 2019a), nor are there sufficient approved treatments to address the (self-)medication needs and increasing number of overdose deaths among stimulant users (Fleming et al., 2020). Thus, as our participants emphasized, access to a safe supply of both opioids and stimulants should be a priority to support self-management among PWUD, especially recognizing that people in our North American setting are managing within the context of an overdose crisis.

Additionally, the value placed on cannabis as self-medication in our data is deserving of attention, for several reasons. First, cannabis was described as a helpful and versatile self-medication among participants for a breadth of health issues. Second, cannabis was not considered to share the complexity of the other drugs that participants used to self-medicate, in particular the need to manage risks of withdrawal, addiction, and overdose. Third, since cannabis was legalized across Canada relatively recently, there may be greater potential to expand its self-medicating benefits into PWUD’s encounters with the healthcare system. In addition to accumulating evidence of cannabis’ therapeutic value across many conditions that are common in this population (Babson et al., 2017; Bhattacharyya et al., 2018; Gibson et al., 2021; Jensen et al., 2015), there is also increasing recognition of its harm reduction potential (Lucas, 2017). Evidence suggests cannabis use reduces the use of drugs like opioids and benzodiazepines (Meng et al., 2021; Purcell et al., 2019). However, the cost of cannabis treatments must also be covered through public drug insurance if socioeconomically marginalized populations are to receive these medical or harm reduction benefits.

Despite several key strengths, including extensive community involvement and a sample showcasing diverse experiences, our study also had a few challenges. First, our sample size was smaller than planned because data collection was interrupted by COVID-19, but we had rich data for each participant thus were able to adjust our analysis focus to ensure the quality of our findings was not hindered. While our sample size was modest and we recommend some caution in interpretation, we found several robust shared patterns with important clinical and theoretical implications, as well as other novel insights that can contribute to hypotheses for further research. Future studies should investigate this topic in other and larger populations and contexts using a variety of methods for further substantiation. Second, even with our community-informed process, the topic was challenging to discuss with participants, resulting in additional time and community engagement for analysis and interpretation. As noted in our results, participants had difficulty focusing on self-management for their chronic health issues and preferred to discuss how they managed their health overall, including their many acute health issues and preventive concerns. However, we recognize that such distinctions are not always easy nor useful to make and believe this is an important consideration for future research. For instance, other self-management studies could either be more explicit about including broad concerns beyond chronic health issues, or impose stricter definitions of terms to facilitate a narrower focus—to determine if these changes lead to new insights. Similarly, given that participants sometimes expressed sentiments indicating they had a wide-ranging perspective on self-care (e.g., including actions like getting a job), future work could also explore various conceptualizations of self-care.

In summary, marginalized PWUD use many self-management strategies to address their chronic health issues and drug use. Chief among these is using non-prescribed drugs to self-medicate their health issues. A host of cognitive and behavioral strategies and creative pursuits are also beneficial, as are seeking out healthcare resources and adhering to healthcare guidance. To “meet people where they are,” especially within the context of the overdose crisis, we recommend providing a safe supply of pharmaceutical-grade drugs to improve the capacity of PWUD to self-manage their health issues. Further, improved access to multidisciplinary pain management, mental health, and substance use treatments are essential, as is access to medical cannabis. We also recommend enhancing opportunities for PWUD to participate in activities that fulfill their creative desires. Existing chronic disease self-management supports are unlikely to fully address the most prominent needs of this population, particularly due to the lack of emphasis on critical topics such as self-medication, harm reduction, and drug use concerns such as withdrawal. Self-management supports for marginalized PWUD should be tailored to include these core needs, while supporting their agency to choose their own self-managing preferences.

## Supplemental Material

sj-pdf-1-qhr-10.1177_10497323221083353 – Supplemental Material for “The Drug Use Unfortunately isn’t all Bad”: Chronic Disease Self-Management Complexity and Strategy Among Marginalized People Who Use Drugs Supplemental Material, sj-pdf-1-qhr-10.1177_10497323221083353 for “The Drug Use Unfortunately isn’t all Bad”: Chronic Disease Self-Management Complexity and Strategy Among Marginalized People Who Use Drugs by Lisa M. Boucher, Esther S. Shoemaker, Clare E. Liddy, Lynne Leonard, Paul A. MacPherson, Justin Presseau, Alana Martin, Dave Pineau, Christine Lalonde, Nic Diliso, Terry Lafleche, Michael Fitzgerald and Claire E. Kendall in Qualitative Health Research

sj-pdf-2-qhr-10.1177_10497323221083353 – Supplemental Material for “The Drug Use Unfortunately isn’t all Bad”: Chronic Disease Self-Management Complexity and Strategy Among Marginalized People Who Use Drugs Supplemental Material, sj-pdf-2-qhr-10.1177_10497323221083353 for “The Drug Use Unfortunately isn’t all Bad”: Chronic Disease Self-Management Complexity and Strategy Among Marginalized People Who Use Drugs by Lisa M. Boucher, Esther S. Shoemaker, Clare E. Liddy, Lynne Leonard, Paul A. MacPherson, Justin Presseau, Alana Martin, Dave Pineau, Christine Lalonde, Nic Diliso, Terry Lafleche, Michael Fitzgerald and Claire E. Kendall in Qualitative Health Research
