# Supplementary material for: “The Drug Use Unfortunately isn’t all Bad”: Chronic Disease Self-Management Complexity and Strategy Among Marginalized People Who Use Drugs
Source: Qual Health Res. 2022 Mar 24;32(6):871–86. doi: 10.1177/10497323221083353 (PMC9189593; doi:10.1177/10497323221083353)
Supplement: sj-pdf-1-qhr-10.1177_10497323221083353 – Supplemental Material for “The Drug Use Unfortunately isn’t all Bad”: Chronic Disease Self-Management Complexity and Strategy Among Marginalized People Who Use Drugs [file sj-pdf-1-qhr-10.1177_10497323221083353.pdf]

<u>Interview Guide</u>

<u>Topic 1: What are the impacts of your health and drug use issues?</u>

1. **So first it would help me to get an overall picture of your mental and physical health. Would you be able to tell me a bit about your health issues and how they impact your life?** *(e.g. symptoms, severity, activities, responsibilities)*
    - **So for this study we're mainly interested in your chronic (or long-term) health issues. Would you tell me which of your health issues you consider to be chronic, and why?**
        - **Do you have any of the most common chronic health issues (e.g. diabetes, heart disease, arthritis, cancer, chronic pain, COPD, STDs, etc.)?**
    - [If not mentioned] **Do you see your drug use as a chronic health issue, and why or why not?**
    - [If not mentioned] **Would you tell me a bit about the types of drugs you use/how you use them, and how it impacts your life?**
    - [If not mentioned] **Which of your chronic health issues would you say is your #1 priority?**

<u>Topic 2: What are you doing to manage the impacts of your chronic health issues?</u>

1. **So we really want to understand what kind of self-care strategies you use to manage the impacts of your chronic health issues. Could you describe what you do on a day to day basis to cope with your chronic health issues?** (e.g. What strategies do you use to handle the negative impacts of these health issues on your daily life?)
    - [If not mentioned] **How does your drug use relate to how you deal with these health issues?** (i.e. How does your drug use affect how you deal with your chronic health issues? And how do your chronic health issues affect your drug use?)
    - **How do you get enough money for your needs (e.g. jobs, selling crafts, dealing, stealing, sex work, panhandling, etc.)?**
        - **How do these activities affect the way you manage your chronic health issues?**
2. **How have the ways you manage your long-term health issues changed over time?** And why do you think they've changed in this/these way(s)?
    - [If not mentioned] **How has your drug use related to these changes over time?** (i.e. How has your drug use affected how you deal with your chronic health issues? And how have your chronic health issues affected your drug use?)
3. **What makes it *harder* for you to manage your long-term health issues, including/or your drug use? What makes it *easier*?** *(e.g. consider factors at multiple levels: intrapersonal, interpersonal, community, organizational, environmental)*

<u>Topic 3: What support do you have to manage the impacts of your chronic health issues?</u>

1. **What support or services have you received to help you manage your chronic health issues?**
    - *I.e. Supports may be anything – formal (e.g. from health or social care systems, including providers, peer workers, community groups) or informal (e.g. from family, friends, peers, neighbours, pets, community activities)*
    - [If not mentioned] How do you feel they have helped/not helped you make improvements at managing your long-term health issues in your daily life?

- [If not mentioned] **How does your drug use relate to receiving this support (or not receiving it)?** (i.e. How does your drug use affect your ability to get these supports? And how do these supports affect your drug use?)

2. **Have you received any specific support or services that were meant to help improve how you *yourself* manage your chronic health issues?**
    - [If yes] Describe what these supports included. How did you find out about them? How did they help/not help you?
    - [If not mentioned] **How does your drug use relate to receiving this support (or not receiving it)?** (i.e. How does your drug use affect your ability to get these supports? And how do these supports affect your drug use?)

Topic 4: What strategies or supports would you like to manage your chronic health issues?

1. **Do you wish to change the ways you manage your long-term health issues?**
    - [If yes] What do you want to do differently, and why?
    - [If not mentioned] **How does your drug use relate to the changes you desire to make?** (i.e. How do you think your drug use would affect your ability to change how you manage your chronic health issues? And how do you think making these changes would affect your drug use?)
2. **What kind of support or services are you *not* getting, but you think would help you manage your chronic health issues *the most*?**
    - *E.g. One-on-one vs. group; peer vs. professional/provider; in-person vs. phone vs. online; formal vs. informal)*
    - [If not mentioned] **How might your drug use relate to getting these supports?** (i.e. How do you think your drug use would affect whether you can get these supports? And how do you think these supports would affect your drug use?)

Questions to wrap up interview:

1. **Which group(s) of people do you *identify* with, or consider to be part of *your* community (or communities)?**
    - **Is there anything about being a part of this/these group(s) that you consider relevant to managing your long-term health issues, including/or your drug use?** [If yes] Can you tell me more about that?
2. **So now that we've discussed a lot of your personal experiences on this topic, I'm wondering if you can tell me what it really *means to you overall* to manage your chronic health issues?**
    - E.g. **Is it important to you to manage your long-term health issues, and why?** (i.e. What are your reasons or motivations?)
    - E.g. **How does managing these health issues fit within your day-to-day life?** (i.e. How much do you think about it? How much of your time does it take? Do you think of it as separate from other things you do, or is it intertwined/connected/linked with everything else? Does it relate to your sense of self/identity?)
3. **What would you say is *the overall relationship between* (managing both) your chronic health issues and your drug use?**
4. Is anything else on this topic important to you that we did not discuss enough during this interview? Anything you thought I would ask you but didn't?
