# Supplementary material for: “The Drug Use Unfortunately isn’t all Bad”: Chronic Disease Self-Management Complexity and Strategy Among Marginalized People Who Use Drugs
Source: Qual Health Res. 2022 Mar 24;32(6):871–86. doi: 10.1177/10497323221083353 (PMC9189593; doi:10.1177/10497323221083353)
Supplement: sj-pdf-2-qhr-10.1177_10497323221083353 – Supplemental Material for “The Drug Use Unfortunately isn’t all Bad”: Chronic Disease Self-Management Complexity and Strategy Among Marginalized People Who Use Drugs [file sj-pdf-2-qhr-10.1177_10497323221083353.pdf]

Supplemental Table 1. Sociodemographic participant characteristics

Characteristic	Participant responses
Age (years)	• Mean = 45 (range = 27-70)
Gender identity	• 7 male<br>• 6 female<br>• 2 other (transgender/non-binary)
Sexual identity	• 10 straight/heterosexual<br>• 5 other (gay/bisexual/pansexual)
Self-identified racial/ethnic group*	• 9 White<br>• 2 Indigenous<br>• 2 White/Indigenous<br>• 2 other
Length of time in Canada	• 13 were born in Canada
First language	• 13 English<br>• 1 French<br>• 1 other
Education (highest level completed)	• 5 Some high school<br>• 2 High school graduate/GED<br>• 6 Some college or university<br>• 2 College or university completed
Training	• 10 had received training from employment or volunteer experiences, including those offered through community organizations (e.g. CPR, cultural competence, kit making, other harm reduction/peer work-related skills)
Income source (last 12 months)*	• 8 Ontario Disability Support Program**<br>• 4 Ontario Works**<br>• 2 Canada Pension Plan Disability<br>• 8 jobs (part-time or casual)<br>• 10 family/friends<br>• 4 sex work<br>• 5 dealing<br>• 3 panhandling<br>• 4 selling handmade items<br>• 6 other street-based income
Housing stability***	Housing stability was mixed among participants. While a few indicated they had adequate permanent housing, the majority expressed some issues with unstable housing (ranging from being unsheltered or in emergency shelter, to facing eviction, to living in rooming houses or inadequate social housing).

*Responses to these questions were not mutually exclusive

**Ontario Disability Support Program and Ontario Works refer to receiving monthly disability or income assistance payments, respectively

***While we did not ask about housing stability in the questionnaire, we summarized data collected in the interviews and provided it here as contextual information

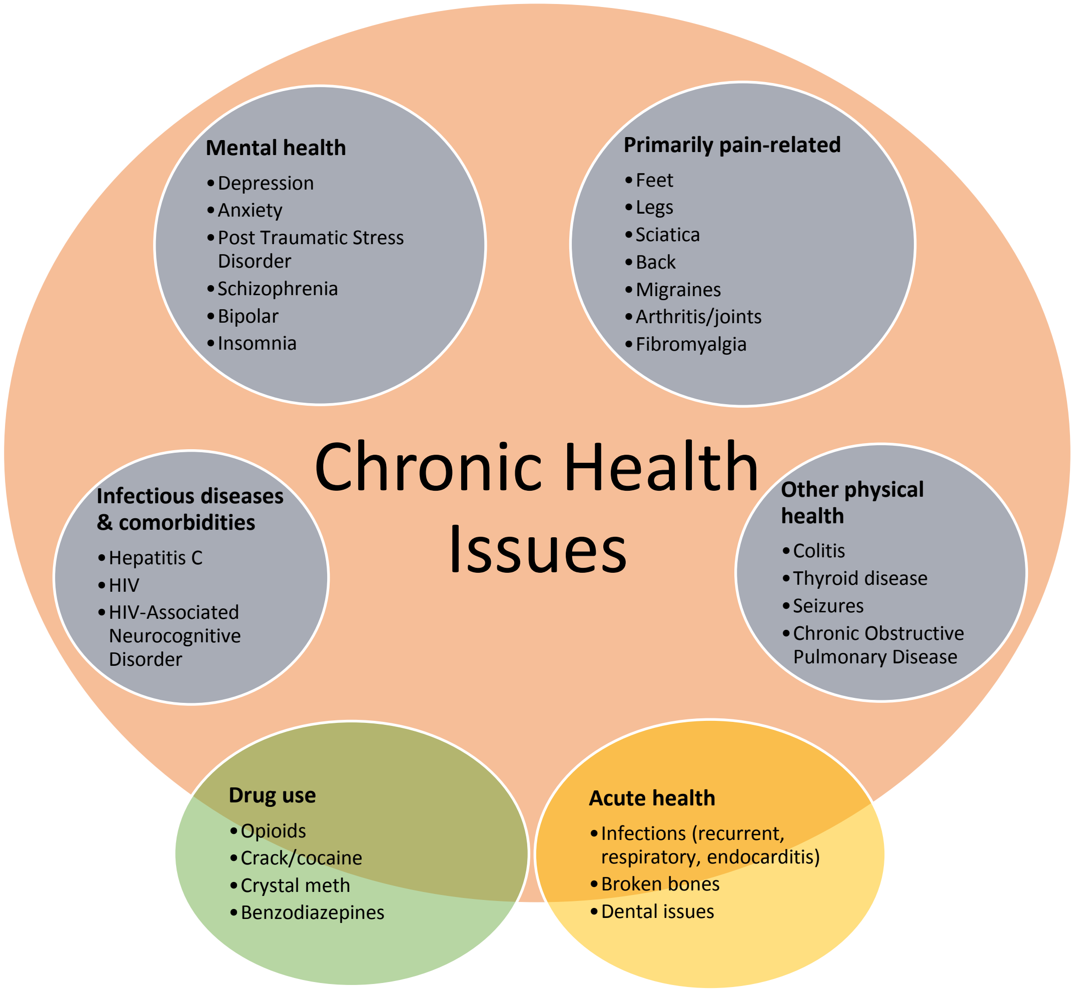

Supplemental Figure 1. Summary of types and examples of participants' overall health issues. The categories of 'Drug use' and 'Acute health' are represented in different colors and only partly overlap with the larger circle because participants considered them to be 'Chronic health issues' in some but not all respects.
